# “Maa. She is Me”: Gendered Emotional Climate in the Making of Therapists in India

**DOI:** 10.1007/s11013-026-10026-z

**Published:** 2026-11-13

**Authors:** Meghna Roy

**Affiliations:** Department of Social Anthropology, University of Oslo, Oslo, Norway

**Keywords:** Counseling training, Gender, Mental health, Mother–daughter relationship, India

## Abstract

In contemporary India, gendered injuries such as affinal violence, son preference, and female feticide may endure within families as mental distress among women. Drawing on twelve months of ethnographic fieldwork at a postgraduate counseling psychology program in Bangalore, this paper examines how trainees’ gendered subjectivities are shaped by growing up within what I call a gendered emotional climate: the durable affective condition that forms as the emotional circumstances of a household, where structural violence is absorbed into kinship, recur and settle over the long arc of growing up. Through a person-centered account of a trainee whose path to becoming a counselor was inseparable from her mother’s history of abuse and chronic distress, I argue that counseling training functions as a form of repair of the emotional climate. It sets in motion the processing of the psychic afterlives of gendered injury and equips trainees to partially rework the gendered emotional climate in their natal households. Training-mandated self-work thus becomes inseparable from filial work. Rather than isolating the subject from family life, counseling may cultivate an ethic of self-making that remains embedded in kinship while partly reworking inherited emotional worlds. This finding has significant implications for clinical understandings of the relationship between gender justice and mental health in post-liberalization India.

## Introduction

On a late winter afternoon, six of us walked back to campus after a hearty lunch, hustling through the narrow lanes crowded with students, rickshaws, and motorbikes in southern Bangalore.^1^ Someone among us said, “Would you want to end up like your mother?” It was common for us—mostly women in our twenties—to have witnessed our mothers or aunts sacrifice their aspirations to take on care responsibilities. Identifying with mothers came with the possibility of identifying with the psychic afterlives of their gendered experiences. The question lingered in the air, charged with ambivalence. Such ambivalence is not incidental to kinship but constitutive of it, since kin relations bind persons through compelling and often contradictory moral claims (Peletz, 2002). For Natasha,^2^ like many of her classmates, this question would become the fulcrum of her self-work—the introspective practice mandated by her postgraduate training in counseling psychology at St Mary’s University. Through her story, this article explores how becoming a counselor in urban India is a deeply relational project of self-making. For women, who comprised the majority of trainees enrolled in the program, the work of self-making was intertwined with filial work.

Since the 1991 economic liberalization, there has been a feminized expansion of counseling and therapeutic self-work in urban centers, where therapy is becoming a recognized practice among the elite and middle classes (Roy, 2025b). According to the erstwhile dean of psychology at St Mary’s, the number of counseling programs in India increased from one to 25 between 2000 and 2016. This made counseling a distinctly generational profession. The significance of this study lies in showing that, in contemporary India, interventions against gender injustice remain incomplete if they do not attend to the psychic afterlives of son preference, affinal vulnerability, and ordinary domestic violence as they are lived within families and carried into the making of future practitioners of care.

The psychological pressures of patrilineal kinship and son preference are everyday conditions of life in much of North India, especially rural Haryana, where Natasha’s mother grew up. Women’s mental distress is rarely a purely individual experience but is instead an “intersubjective” phenomenon deeply rooted in the domestic sphere and thus transmitted into the felt emotional climate of the household. A primary idiom of distress is “tension” (tenshun), which serves as a dimensional category mediating between external structural stressors—such as poverty, debt, or family conflict—and internal pathological states (Simpson, 2026, 1–26; Weaver & Karasz, 2022). For many women, this “tension” reflects the crushing weight of affinal vulnerability, a precarious social positioning where a wife’s security in her husband’s home is contingent upon her ability to "adjust" to the demands of her in-laws (Chua, 2013). In the wake of violent events like female infanticide and feticide, the spectral traces of gendered wounds seep into the everyday, as the afterlives of violence often do (Chatterji & Mehta, 2019; Das, 2006).

Pinto observes that affinal vulnerability—the inherent precariousness of a woman’s position in her husband’s household—remains a primary site of mental health crises, leading some scholars to characterize hysteria in India as "the wife’s disease." In many North Indian contexts, in-marrying daughters-in-law are often perceived as potential threats to the moral coherence of the patrilineage, making them uniquely vulnerable to psychological distress or “madness.” Symptoms such as “chakkar” (giddiness), “dissociative stupors,” or “clenched teeth” are frequently read by clinicians as manifestations of agency gone awry within the family. For many women, madness is a manifestation of the fragility of kinship bonds, where the fear of persistent dislocation and the potential loss of children are woven into the normal, if heightened, stresses of marital life. Women are forced to inhabit a psychological space where freedom can bleed into abandonment, and care is often inseparable from constraint (Pinto, 2014). These psychological spaces are charged with emotional climates that persist over long periods. To the married woman, it could be most of her married life, and to her children, most of their life at home may be characterized by such a persistent climate of distress.

The domestic environment in India represents a unique “zone of social abandonment” for the severely mentally ill. Unlike the Western model, where abandonment most often manifests on the street, in India, disordered individuals are frequently “hidden” within the family home to preserve family honor (*izzat*) (Marrow & Luhrmann, 2012). As we see in Jhumpa Lahiri’s (2019 [1999]) prose, this confinement is often an extension of “kin-work,” where the family struggles to inhabit a damaged world of broken bonds. Antiviolence counseling in Northern India operates by seeking to reorder, rather than eliminate, the hierarchical dependencies and interconnections that define a woman’s position within her family (Kowalski, 2016). With few emotional outlets available, the child may serve as the object of the mother’s unfulfilled desires; this can cause the child to feel like a “savior” for the mother, potentially transforming the “good mother” into a “bad mother” who is perceived as overwhelming or devouring (Kakar, 2012, 89–92). Many of the counseling trainees I met during fieldwork were admittedly predisposed to a savior role in interpersonal relationships.

Most trainees enrolled in counseling training programs in India are women. Sociologist Anne Gagnant de Weck observes that the vastly disproportionate number of women enrolling in psychotherapeutic training and practicing as therapists is driven by a complex interplay of cultural stereotyping, economic expectations, and the unique gendered tensions of India’s transitioning society, making counseling a feminized profession in the subcontinent. Psychology is widely stereotyped as a feminine pursuit, thus making it difficult for men, who must bear the breadwinner burden, to pursue it. Psychotherapy, being a highly flexible practice, allows women (often the trailing spouse in a heterosexual marriage) to continue practicing it even as they move to accommodate their husbands’ careers in other fields like engineering, medicine, and corporate establishments. When women attempt to claim personal autonomy, choice, or happiness, it frequently triggers intense moral panics and family conflicts. They find in psychotherapeutic concepts a vital “laboratory” and a vocabulary to negotiate their own desire for individual agency, make sense of emotional distress, and create a space of their own (Gagnant de Weck, 2023). Course-mandated self-work partially converts gendered injury among trainees into internal resources.

This paper focuses on the effects of such internal resources, developed through training, on the emotional climate of mothering in the natal households of future mental health practitioners. According to psychoanalyst Sudhir Kakar, the mother helps the infant “learn to deal with anxiety without feeling devastated, and to temper and manage the inevitable feelings of frustration and anger” (2012, 55); for this reason, he urges anthropologists and psychologists to attend not simply to discrete maternal acts but to the “total emotional climate, the gestalt, of mothering” (2012, 55–56). The “good mother” in an Indian setting is normatively expected to be a nurturing, fear-dispelling presence. Her “hallowed presence” provides life-giving reassurance to the child, forming a bedrock of basic trust in the environment. The emotional climate of mothering is thus the mother’s presence as a whole human being, rather than just a provider of physical needs such as feeding or weaning (Kakar, 2012). This formulation is useful because it shifts analytic gaze away from a distressed mother as an isolated source of psychic injury and toward the larger affective field in which mother and child come to inhabit one another’s worlds. It also draws attention to the transmission of gendered harm in the form of a long-term felt condition for children.

In this paper, I extend Kakar’s formulation of the dyadic emotional climate between mother and child to what I call a *gendered emotional climate*. It refers to the enduring affective condition in which the mother’s subordinate status in a patrilineal setting shapes the emotional circumstances of the child’s development. This framing acknowledges that the mother’s capacity to soothe her child is associated with her position and overall gender norms in the household. Gendered wounds are often lived as diagnosed and undiagnosed forms of mental distress in mothers within families in South Asia (Clarke et al., 2014; Das et al., 2012; Nayar & Mehrotra, 2015). Female counseling trainees who grew up in difficult emotional climates related to the devaluation of daughters often share the suffering of their mothers. For the daughter, the mother is a significant source of the predominant cultural norms of femininity.

Anthropological and sociological scholarship has long cautioned that the spread of psy-knowledge individualizes structural problems, pathologizes ordinary distress, and produces governable, neoliberal selves (Furedi, 2004; Illouz, 2008; Rose, 2005). In Indian debates, these worries (Gooptu, 2013) converge with Hindu-nationalist critiques of psy-disciplines as solvents of collective life (Aggarwal, 2025). These critiques have force, but they often target therapeutic discourse rather than the neoliberal structures within which it operates, and risk obscuring the ways psy-concepts can also enable subjects to name, rather than internalize, the harms those structures inflict (Funahashi, 2023; Kitanaka, 2012). Vorhölter (2022) argues for a “more-than-critique-anthropology” in her ethnography of the emergence of psychotherapy in Uganda; this approach takes practitioners’ visions seriously as one valid analytical perspective among others. Besides Vorhölter, I follow Duncan (2018) in treating psy-globalization as a dual process: globalizing psy-ideas are transformative for and transformed by the contexts they enter. They articulate with political–economic projects and, at the same time, open sites where those projects can be commented upon and reworked.

I argue that training reframes maternal suffering from individual maternal failure (as mental illness often gets framed) to the psychic consequences of gendered violence, both symbolic (Morgan & Björkert, 2006) and physical. During mandated “self-work,” which is a key part of training as a counselor, childhood emotional climate becomes both an object of interpretation and an inner resource for care with transformative potential. Trainees are offered a framework for understanding the mother’s actions as the effects of gendered suffering rather than individual failure. Training thus cultivates a therapeutic self by transforming gendered subjectivity into a capacity for listening, reframing, and relational repair among kin. After discussing the method and context of my study, I will discuss the climate of mothering that Natasha experienced as it emerged during training. Then I will discuss how she worked on herself in relation to her mother during the program. The program facilitated new emotional climates both within and beyond the classroom. Following this, I will demonstrate the salience of focusing on mother–daughter relationships in patrilocal and patrilineal contexts marked by volatile emotions as a possible means of changing the gendered emotional climate of mothering. I conclude with clinical implications of the concept of emotional climate beyond my immediate field site and policy recommendations to address female feticide and infanticide.

## Method and Context

My primary ethnographic field site was the postgraduate counseling training program of a leading private university in Bangalore, which I refer to as ‘St Mary’s University’ (hereafter ‘St Mary’s’), managed by an indigenous congregation.^3^ I participated in pedagogical activities, such as classes, conferences, seminars, and therapy^4^ simulations, as any student enrolled in the program would. My research design was assessed by the Norwegian Data Protection Services (Sikt) as having a legal basis to possess personal data. According to Article 6(1)(e) of the General Data Protection Regulation, the legal basis of processing personal data was the data subject’s consent.

My position oscillated between an observer who participated and a participant who observed. I conducted semi-structured interviews with sixteen counseling trainees and a few trainers. I maintained confidentiality across these relationships, since my role shifted between confidant to students and collaborator with instructors. While this analysis draws on these interviews, it follows a person-centered ethnographic approach that privileges depth over representational coverage (see also Hollan 1997). Natasha’s trajectory is examined in detail rather than abstracted from the interviews because it offers a particularly rich window into the processes of self-work, relational repair, and reinterpretation of maternal suffering that recur across multiple interviews (of trainees like Anirudh, Snigdha, Roshni, and Yamini mentioned in this paper). Natasha’s retrospective narratives, part of which were her accounts of her mother, were my objects of analysis. These narratives were an effect of the program, as it helped her interpret her relationship with herself and her mother. I make no claim to her mother Sarika’s experience, to which I have no independent access. I conducted member checking with Natasha, a key contributor to the data presented in this paper. I also conducted interviews with counselors (graduates of St Mary’s and other programs in major cities) who had recently started practice.

Only four of the 57 trainees in the cohort identified themselves as men, while the rest were women. All trainees, barring two outliers (who were 25 and 26), were between 20 and 22 years of age. The gendered nature of training’s effects on counselors’ lives led me to examine the continuum between being a daughter and being a counselor. This focus on the daughter–counselor continuum reflects the feminized composition of the cohort; how the same self-work bears on the gendered subjectivities of the male trainees—their work as men—lies beyond the scope of this article. It is a limitation and promising direction I flag for future research. Further, training operates in the interpretive and relational register, while leaving the consequential material conditions of gender injustice almost unchanged.

I lived alone in a one-bedroom rental apartment close to campus during my year of fieldwork. Classmates from St Mary’s often dropped by. To most of them, who lived in paying-guest facilities, my place, admittedly, felt like “home,” where they could spend time with their friends from class, away from the watchful eyes of hostel wardens or the invasive presence of roommates. Sometimes we had extended discussions, parties, movie nights, or we cooked together. Living alone near campus enabled forms of intimacy and conversation that would not have been possible in the program’s formal spaces. My apartment became a site of fieldwork, where trainees often continued the reflective and relational exercises initiated in class. It let me live with the inner worlds of my interlocutors, which was key to my primary interest—self-making practices in counseling training. Opening my home to trainees allowed me to notice the self-work that happened beyond institutional settings.

I approached Natasha’s story as a person-centered (Hollan, 2005; Levy, 1993) subjective account situated in a moral and historical field. This approach enables a researcher to take the symbolic and psychic labor of self-making seriously—how people work through past injuries, not in isolation but with relational responsibility toward others. Rather than a Western import or a local distortion, therapy emerged as a site where gradual, recursive self-making unfolds. A person-centered approach to ethnography allows us to dwell on the contradictions that emerge in ethical experiments in everyday life, rather than collapsing them into normative theories of personhood. I understand the core assumption of this method as:

“Recognizing the multiplicity of human conditions, we affirm that our subjectivities and the moral processes in which we engage are forever in flux—[…] As our worlds change, […] they remake our most intimate inner processes: emotion, cognitive style, memory, our deepest sense of self.” (Kleinman & Fitz-Henry, 2007, 55)

Person-centered ethnography is thus a powerful technique to analyze a process as intimate as the interlocutor’s metamorphosis of self.

My unit of analysis is the self-work of a trainee, or the intentional process of discerning and reflecting upon one’s own thoughts, emotions, and behavioral patterns during training. In its attention to how practitioners are formed, mine is an ethnography of clinical training in the sense of Luhrmann (2001), whose concern is with what young clinicians learn to notice and how they come to notice it rather than with the validity of any particular mode of treatment. Any ethnographic exploration of subjectivity needs to be linked to gender (Good et al., 2008) under prevailing normative orders. In my fieldwork, the practice of self-work is improvised within constraints—specifically, a patrilineal kinship system and the symbolic violence of rejecting female children. This allows for both continuity and change.

## Gendered Emotional Climate

Natasha read out of her reflective essay on family in a Professional Development^5^ (PD, or process development) class: “*To understand who I am, we need to understand [...] my mother*.” [emphasis mine] She urged us to picture her mother, Sarika, as “a woman rocking a summer dress in her fifties”—a bold sartorial choice for a Haryanvi woman living with her in-laws in North India. This image contrasts Sarika’s younger self, “shipped off” according to Natasha, as a teenager from her natal village to her affinal kin in New Delhi, into a conservative multigenerational Baniya^6^ household, where she endured years of physical and emotional abuse from the matriarch, her mother-in-law, until she, along with her husband and children, moved out of Natasha’s ancestral home only a few years ago. The mother-in-law’s grip persisted over the emotional climate in the ancestral home, and Sarika still bore traces of the abuse. However, she was able to begin altering her emotional climate as a mother. By this time, Natasha was an adult and intimately familiar with the complex relationship of a bride with the mother-in-law in a climate of son preference.

Marriage is central to the identity of women in South Asia, as singlehood and divorce continue to be taboo (Lamb, 2018). The transition from natal to affinal household is riddled with hurdles for a bride in a patrilocal system. Traditionally, it has been challenging for most brides to find allies in their mothers-in-law. Incorporated as the 'gift' from bride-givers to bride-takers, the in-marrying bride enters as subordinate to a mother-in-law whose seniority confers authority, owing her service and deference, thus producing durable affinal asymmetries (Dube, 1988; Karve, 1953; Vatuk, 1975). However, this relationship is fluid, strategic, and rooted in material circumstances rather than timeless norms (Vera-Sanso, 1999). For instance, per Natasha’s account, her mother, Sarika, was able to create a condition free of fear and subordination after moving out of her mother-in-law’s household; an emotional climate that had long stifled her and her dependents began to change. Leaving the house was a change in material conditions that was perhaps as effective as, or even more effective than, any reinterpretation that psychotherapy offered.

Sarika’s subjectivity was shaped by long-standing patriarchal norms and gendered violence, all of which seeped into Natasha’s and her siblings’ affective experience of their mother. Natasha’s emotional climate growing up was volatile. Her father and sister were a passing source of stability. After the sudden demise of Sarika’s brother when Natasha was in fourth grade, Sarika entered a prolonged period of grief, often withdrawing into herself. The memory of her mother crying uncontrollably upon the children’s return from school remains etched in her mind as a ‘core memory,’^7^ shaping Natasha’s inner world. Natasha recalled this as a time when her mother “was not herself.” This observation, grounded in her training, was not merely descriptive. For Natasha, “self” is a term infused with meaning—both a psychological construct and a professional tool, as the self is the primary instrument counselors use. Her mother’s fractured sense of self deeply shaped her own; Natasha admittedly had an “anxious attachment style” that, in her opinion, impeded her success in romantic relationships.

Natasha was far from the favored child: her brother Shashank, born with a speech impairment, received greater affection from Sarika, while Natasha felt she had to earn her mother’s love. Her sister Koyel—who unwound with Natasha through “emergency dance parties” during challenging times—served as a quasi-maternal figure, someone she even once wished on Mother’s Day. Sarika would have birthed a girl child when she conceived for a third time if not for the forced termination of her fetus following sex determination,^8^ which is an illegal but existing practice in several regions, especially northern and northwestern India. Sarika managed to keep the sex of her fetus from affinal kin for the first three months of her fourth pregnancy. That is how she was able to carry Natasha to term. Natasha learned about her family’s history of female feticide as an adolescent when, in a fit of rage, her mother retorted, “I should have aborted you when I could.” The remark left Natasha with a lasting feeling of rejection. By the end of Natasha’s reflective essay on family, which recounted the above details, Sarika’s emotional climate of mothering had moved many in the training cohort to tears. Sarika was both Natasha’s primary object of identification and a sustained source of emotional wounding.

Natasha brought glimpses of her mother’s various experiences of gendered injury into the classroom by sharing that her mother had a neurotic personality. According to the counseling curriculum, neuroticism, a personality trait rather than a mental health condition, is described as a frequent inclination toward negative emotions like anxiety, hopelessness, and sudden mood fluctuations. Sarika’s distress was inseparable from her subordinate position within the kinship system. Much of her distress may have been a form of coping with personal grief in a hostile affinal ecology of son preference. Take this verse from a Haryanvi folk song celebrating son preference, for example: “*Suhagan gaavein, putar khilavein* (the married women who sing receive sons).” (Toor, 2021).

**Fig. 1:**
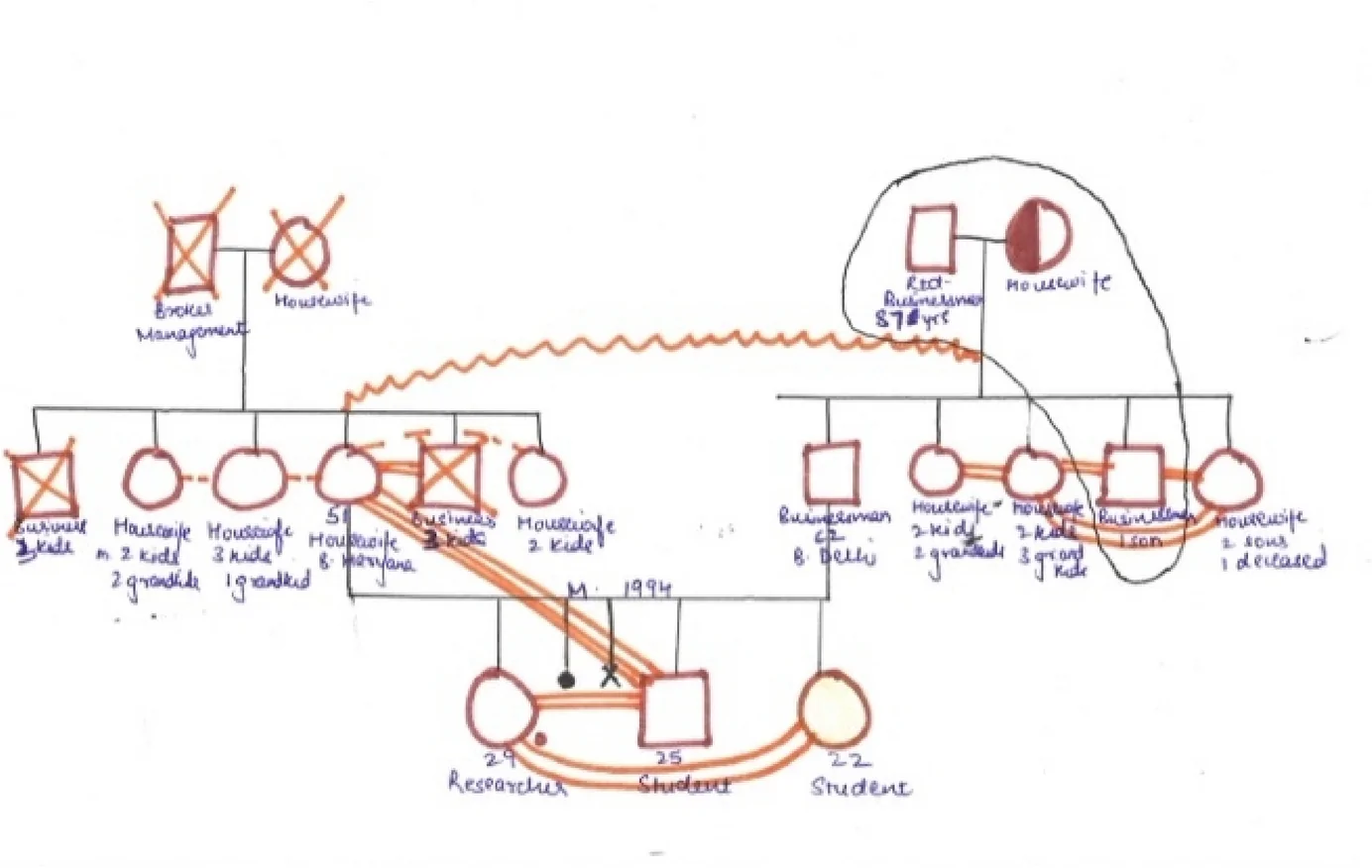
Natasha's genogram, a visual representation of a person’s family tree. The dot signifies Sarika’s miscarriage, while the cross stands for her abortion

Natasha’s eyes beam when she recounts how her father gave up karate after his first daughter was born. His karate practice involved the risk of physical injury. Natasha’s relationship with masculinity has been rough despite sharing a healthy relationship with her father. When Natasha was eight, a man masturbated to her in public while she was returning from school with her mother by a rickshaw—unfortunately, not an uncommon form of sexual harassment in New Delhi. At 12, her cousin sexually harassed her. Her ex-boyfriend, during her first experience of romantic touch, “realized” he is asexual after their first act of physical intimacy. Hence, touch has been a complex subject for her. Self-aware as most trainees were, Natasha associated her own anxious attachment style with the contradictory forms of masculinity she had experienced. She “sabotaged” her romantic relationships in the past—a pattern she is working on in her current relationship with Suman Lokhande, a fellow trainee, who, Natasha believes, “is more empathic than” her. Natasha and Sarika had both experienced emotional challenges that took sustained, deliberate effort to be overcome (Fig. 1).

**Fig. 2:**
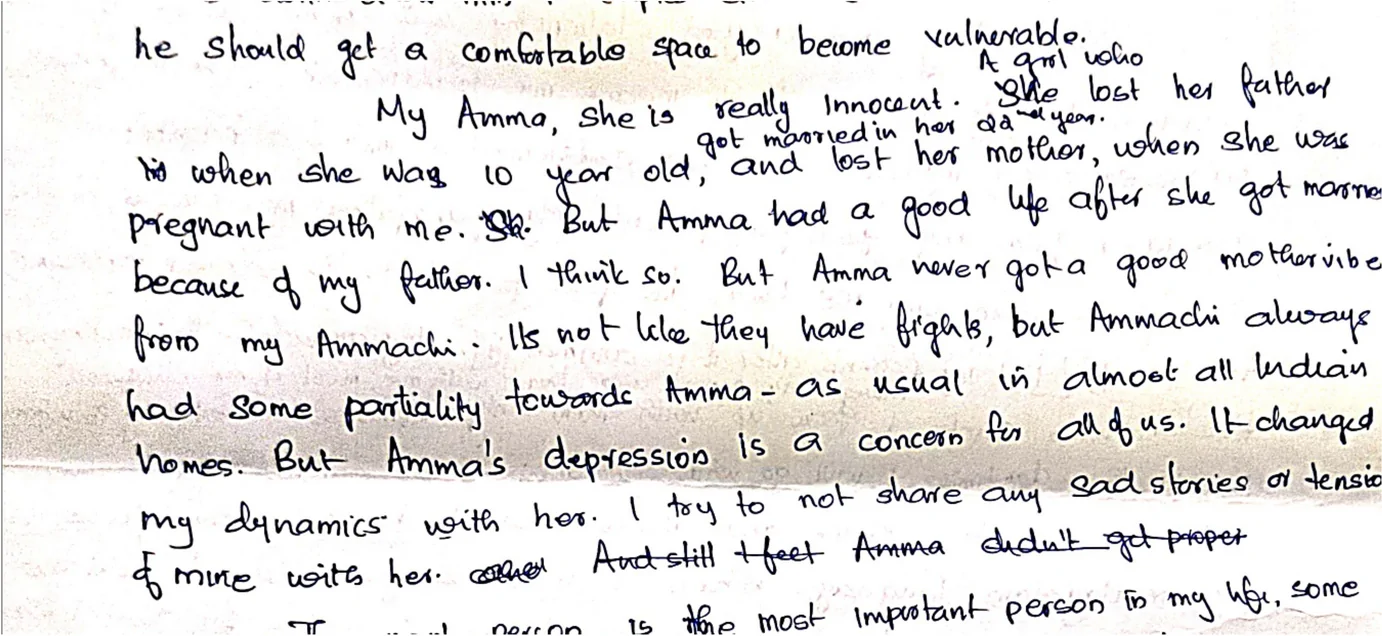
This paragraph about Susan’s mother is from her reflective essay about family for the process group

Natasha was no exception among her cohort in terms of intergenerational wounds. The following is an excerpt from her peer Susan Aleyamma Cherian’s reflection:

Susan’s *amma* (mother), besides receiving differential treatment from Susan’s *ammachi* (paternal grandmother in this case, although the term is also used to address mother and maternal grandmother among Malayali), was depressed and had lost her parents early in her life. In her essay, Susan is explicit about how her *amma*’s distress had an impact on the relationship between them. All her life, Susan was cautious about sharing her “sad stories” with her *amma* so as not to aggravate her suffering or contaminate the prevailing gendered emotional climate that the household was accustomed to. She described her *ammachi’*s differential treatment of her mother “as usual in almost all Indian homes” (Fig. 2). The persistent tension in this relationship is a common theme in South Asian tales and folklore. For instance, the lyrics of a Haryanvi folk song—sung on the festival *Teej* in the month of *Saaman* when married women sing and swing in groups awaiting their brothers’ *kothli* (gifts)—loosely translate to the following:

*Bed batade sasu reshmi ri—* (Plait a silken rope, dear mother-in-law—)

*Aari ghalwade baag ke beech,* (In the middle of the garden…)

*Baag jhulan heri jaan de ri.* (…get it hung.)

*Kun je ghale teri jhool ne he* (To swing in the garden, let me go!) […]

*Bed batadu bahu reshmi he—* (Plait a silken rope I can, dear daughter-in-law—)

*Aaye ghalwadu mahal ke beech.* (In the middle of the mansion, I can get it hung.)

*Baag jhulan ki to taal se he.* (To swing in the garden, you cannot go!)

*Lal gaya pardes me he,* (My dear son has gone to a distant land.)

*Aaye bahu log karenge takraar.* (O daughter-in-law, people around will create a scene.)

*Baag jhulan ki to taal se he.* (To swing in the garden, you cannot go!)

(Toor, 2021)

Sarika was not simply the source of distress; she was herself immersed in and formed by a climate of subordination that exceeded her (Bourgois, 2003, 284–285). In Natasha’s case, what mattered was not only Sarika’s speech, moods, or decisions taken in isolation, but the total emotional climate in which mothering took place: affinal violence, unresolved grief, son preference, sibling differentiation, sexual threat, and the everyday vulnerability of being a daughter in a patriarchal kinship order.

Following Jenkins (2025), emotion need not be treated as a process sealed within the 'box' of the psyche; it can instead be understood as an atmosphere that traverses the space and time of bodily longing, memory, gendered power relations, and psychic conflict. Feeling, in this view, is not only something that radiates outward from a bounded self, but a shared socioemotional space of power and position to which kin become attuned. This is why Natasha’s childhood is better understood not as life with a “difficult mother” but as life inside a gendered emotional climate in which her mother’s suffering, her grandmother’s authority, her father’s relative tenderness, her brother’s favored status, and the spectral presence of the aborted female foetus all coexisted and pressed upon her. As Wu (2025) notes in the context of China, intimate feelings are inseparable from wider structures: what was lived as volatility or neuroticism within the family was intertwined with longer histories of patriarchy, affinal domination, and daughter devaluation.

## Training as Relational Repair

On a November evening, Natasha and her peers, Anirudh, Snigdha, and Roshni, gathered in my living room. After cooking, dining, doing the dishes, and intermittently using the fan as the temperature fluctuated, we sat around my low round coffee table. The conversation drifted gradually toward marriage. Marriage was no longer a compulsion for most trainees, as it had been for their parents, especially their mothers. So, when relatives asked when Koyel would get married, their parents defended her. Natasha’s mum often said, “She is happy; how many of us can say that about our lives?” After recounting this, Natasha remarked that the generation before her “just got married because they had to and stayed married because they had to.” “All Indian families,” remarked Anirudh. What appear as low divorce rates in India are less signs of marital stability than outcomes of dense moral, economic, and gendered constraints that make leaving both difficult and consequential (Pinto, 2014). Together, the trainees were beginning to attune to feelings that eventually settled as shared feelings that pervaded their emotional climates in Bangalore. They recognized and named patterns of the reproduction of gendered injuries. Training signaled that subjects with some agency could transform the emotional climate around them.

Natasha and Snigdha shared that they were disturbed by their respective grandmothers' opinions. Both normalized domestic violence against wives by husbands. Natasha was uncomfortable with her *dadi* (paternal grandmother) eating from *dadu’s* (paternal grandfather’s) used plate after he had finished his meal. Natasha “protested a hundred times” against her *dadi’s* practice. Snigdha noted how when she first got her period, *maa* (meaning mother, a term she used to address her *dadi*) commanded her to maintain rules of purity and pollution, for instance, not to enter the kitchen when menstruating. Despite acting out of habitual disposition in matters concerning the self and the daughters-in-law, grandmothers were affectionate toward their grandchildren. The emotional patterns reproduced at specific spaces (Reckwitz, 2012), in this case, the patrilocal household, sustained both adoration and resentment in the gendered emotional climate of growing up.

The shift in thinking about family life, shaped by structural constraints, was consequential. It helped trainees see their mothers and grandmothers less as troubled individuals and more as inhabitants of a gendered emotional climate: the felt condition of constraint, grief, and subordination that patrilineal kinship produces inside domestic space. For Natasha, becoming a counselor involved confronting intergenerational family climates of violence and suffering. Like many of her classmates, Natasha reconfigured relational subjectivities intertwined with modes of kinship and care. She tapped into her potential to *break the pattern*, referring, among other things, to moral injuries to the gendered self (in this case, Natasha and her mother) in patrilocal emotional climates. Breaking the pattern here does not mean severing ties or breaking up families. It refers to the (partial) breaking of relationships between individuals. It breaks away not from family members but from ways of relating to them that sustain moral injuries, and what is an emotional climate if not a sustained mode of relating to the other?

Rather than merely learning therapeutic techniques, trainees are expected to undertake sustained self-work. Not only do these practices build skill, but they also cultivate specific ethical subjectivities characterized by empathy, reflexivity, and emotional regulation. At St Mary’s, trainees were theoretically trained in empathy and its various types, including additive and subtractive empathy. Instructors in their skills labs urged students to practice empathy in interactions with clients. The effects of self-work at St Mary's went beyond individual trainees. By naming the emotional climates of their upbringing—the volatility, the grief, the gendered silences—trainees began to alter those conditions, not only for themselves, but for the kin they carried into the room. The program facilitated new emotional circumstances, or climates, both within and beyond the classroom. These climates mattered because, sustained and repeated, they began to recompose the gendered emotional climate that trainees carried from their natal households. I foreground this durable change as the work of relational repair; more reflexive practice and more livable intimate lives follow from it as secondary effects. These effects were uneven but observable across the cohort.

In the PD batch I attended, Bella, an American instructor from Texas, said, “We wouldn’t be in this profession if we weren’t empathetic^9^ people.” The person-centered argument that counselors must be empathetic was implied, but trainees were taught how to express empathy as counselors to help heal clients. I understand this ineffable “effect”—not the feel of a single session but the durable affective baseline that sustained therapy works to shift—as emotional climate. Self-work emboldened Natasha to cultivate a self who would be open to the possibility of a romantic relationship with a man. Training is a moral laboratory (Mattingly, 2014) that lets Natasha try out new ways of being a daughter, this time with the possibility of becoming a therapeutic self. It is her renewed attempt at being her mother’s daughter, in turn creating a second chance at mothering for her mother. Their deeply moral and relational process of self-making, in the words of Veena Das (2006, 61), is “the limit at which the self separates into that which can be destroyed and that which must endure.” What does storying do for the trainees? A practitioner’s painful memories are not obstacles to care but its very medium (Chapman, 2025)—a blurring of patient and healer that, in Natasha's case, extended to her mother.

Natasha’s self-work did not stop at the mother–daughter relation. While self-work did not dissolve the limits her family imposed on her sexuality, for instance, training gave Natasha a means to live within these constraints differently. Her intercaste relationship with Suman—kept hidden from her biological family, navigated through the borrowed domestic space of my apartment—represented a parallel practice of kinning (Howell, 2003), one in which the values learned in counseling replaced caste as the organizing logic of intimacy. Drawing boundaries here did not mean severing family ties but gradually choosing proximity to some relations and distance from others with an intentionality that training had made possible. Suman and Natasha delicately navigated the nuances of intimacy and the space between them.

Over years of therapy, Natasha partially reshaped her selfhood in the directions she wanted. She continues to negotiate her way toward the person she wants to be—the one most aligned with her vision of the future—and, as her training recommends, an *authentic* version of herself. For instance, she overcame her fear of men and went on to see a male therapist. It was a milestone in her personal journey to becoming a counselor. Her emotional climate around men who were not her father was warming up. She believed she could look back on her choice of a male counselor as evidence of practicing what she “preaches” when she would encourage a future client to overcome fears. Drawing parallels with confessional modes, trainees are required to articulate inner truths to supervisors and peers, rendering their emotional climates alterable. The measures of such self-cultivation may not conform to normative expectations of particular gendered, caste, or class relations. Thus, counseling training is not simply professional education; it is a unique disciplinary and ethical project that recognizes trainees as both long-term subjects and agents of relational repair.

## Emergent Emotional Climates in the Household

Natasha proudly recounts how Sarika, who never attended college, has three children with postgraduate degrees. To Natasha, her siblings’ formal education is a step in the right direction. Despite early injuries, Natasha’s self-work was saturated with her mother’s presence. She wrote,

*“Maa. She is me. Or I am her. By some unexplainable science, we have the same heart.”*

This is not a description crafted over “a weekend of introspection” of what growing up in a gendered emotional climate does: it produces a self inseparable from the relational conditions of its formation. Neither was Natasha’s identification with Sarika an evidence of failed separation. As Martin (2025a) argues, the infant does come to develop a sense of being separate, but this differentiation is never final or complete; it remains a form of separateness constituted in relation to the caregiver. Natasha’s self-work thus did not require detaching from her mother so much as renegotiating the terms of that relation. Natasha and Sarika do not simply share memories or traits; they share an emotional climate to which they were both porous, one that women in patrilocality are especially susceptible to. This confluence between Natasha’s self and her mother’s self is generative. It shows that Natasha moves between unity and separation, drawing the boundaries of selfhood to varying degrees and in different contexts. Natasha’s therapeutic engagement did not aim to detach from Sarika but, in counseling terms, to ‘re-story’ their intertwined lives. Thus, it redrew how they related to each other, and how strongly the spectral traces of an unborn girl would resonate between them.

Natasha addressed the learned victimhood in her mother. To her, Sarika was at once an aspirational ideal and a cautionary tale. She respected her mother’s resilience despite the repeated hardships she endured. She explained to Sarika the possibilities within her kinship framework, considering her husband and mother-in-law, both of whom were placed higher in the kinship hierarchy than she was. She did not have to sever ties with her in-laws or lead a family life isolated from her sense of self. She learned to live with ambiguity, not by detaching from kinship, but by relating differently within it. If kinship is a practice, then it is both structured and structuring (Bourdieu, 1977; Lahire, 2020). The grammar of North Indian kinship^10^ is the structure, while a relationship’s evolving emotional dispositions are the forces that structure it.

A parallel pattern surfaced in my interview with Yamini, a 22-year-old trainee from Tamil Nadu. She described teaching her mother techniques she had learned in class. “I have literally trained my mother to be another counselor,” she said. Yamini also reported that her mother’s relationships with her own mother-in-law and husband had eased as her mother began considering others’ perspectives in ways she had not previously. The reach of this repair was confined to Yamini’s mother: “I've tried my dad…did not work. My sister…did not work. Grandfather is like an extreme level, can’t change him at all.” Yamini's mother, too, was unsettled rather than simply gladdened, telling Yamini, “I have changed so much because of you… I don't know whether I should be happy or sad about it.” The gendered emotional climate of mothering, then, could be reworked across regional kinship grammars, but this reconfiguration was uneven, and not always experienced as welcome by those it touched.

Repairing a gendered emotional climate does not mean dismantling the kinship structure that generated it. The present terms governing relations among household members vary depending on how one person addresses another, what can be said aloud, and, most importantly, what is classified as an individual member’s failing. It means durably reworking the relations and habits through which the emotional climate is sustained. Much of Sarika’s affliction, which was a symbol of a disordered kinship, was mitigated by reworking familial ties like pieces of a puzzle that fit within the board, not by isolating or trying to fit a piece into another puzzle altogether. Therapeutic repair is set in motion by reinforcing the value of care back into kin networks (Kowalski, 2016). Similarly, Natasha tried to rework her inherited wounds into future-oriented forms of care. Her emotional labor in this repair work was facilitated by course-mandated self-work, which included at least 10 sessions of personal therapy and various reflective exercises, such as journaling and instructor-assigned introspective dialogs. By addressing the fractures in her selfhood, Natasha addressed the fracture in her mother’s sense of self and their mutual network of kin. For instance, she psycho-educated her family about how to navigate lingering interpersonal feelings. As the self is relationally constructed (Csordas, 1990), so is its fracture and, in turn, repair.

As she was setting up her practice after graduating from the program, Natasha decided to name it after her mother. Sarika’s place within kinship networks had been elevated. Besides her children’s education, her new style of clothing recovered her sense of self. Her exact date of birth was unknown to her family due to discrepancies among her identity documents. Earlier, the discrepancy had been a sign of parental neglect toward a girl child. Since her husband and adult children began cutting cakes to celebrate her birthday on more than one day each year, the household came to associate a feeling of lightness with Sarika’s birthday. She was beginning to renarrativize her life, and that had a definitive bearing on her immediate family. Even in its oblique and partial form, storytelling can still function as moral agency, for what Myers (2024) calls autobiographical power lies in retaining some authorship over one’s life story under conditions that threaten to erase it.

Women’s narratives of loss can reflect both structural marginalization and intimate, embodied experiences of sorrow. Sarika’s revelation about feticide to Natasha in a fit of rage was an oblique reference to her continued grief. Her general silence about feticide and the fear of it may have been an everyday gesture of her survival strategy under threatening circumstances (Das, 2006). Mattingly (2010) discusses narrative restraint and fragmented storytelling in contexts of medical suffering, illustrating how people navigate grief not through linear catharsis but through partial, situated articulations. Natasha says that both sides of her family had practiced feticide. The cycle of grief, loss, and fear had ended for their immediate family that shared a household. The gendered emotional climate under the shadow of feticide that had persisted in their family over generations was now about to change. Natasha’s training period inhabited this threshold moment.

Growing up as a witness to the violence meted out to her mother, Natasha tried to rebuild her sense of self and, in turn, her intersubjective relationship with significant others. As Veena Das (2006) notes, acts of violence, besides being witnessed, are reconfigured as everyday life unravels, and new cultural worlds are created. For instance, Raheja and Gold (1994, 20) observe,

“[…] patriliny, hierarchy, female subordination, and so on are seen not as aspects of a fixed and reified cultural system but as strategically invoked idioms in an ongoing negotiation of personhood and relationship that, like proverbial utterances, may often be countered with other contrasting idioms that evaluate the situation at hand in quite different moral terms.”

This is precisely what the concept of gendered emotional climate captures: not a fixed structure of oppression but a lived, shifting condition that can be entered differently once its conditions are named. Natasha’s refusals—of early marriage, of her family’s choice of groom, of Sarika’s emotional climate of mothering—altered the emotions that characterized their shared everyday. By refusing to marry early (by her mid-twenties) a groom chosen by her family, by venturing out of her comfortable life in Delhi, by pursuing a master’s degree, and by tending to her feminine side—to the Natasha who likes to play with dolls, who likes pink and to decorate her room with a vision board—she learned to live on her own terms. In psychoanalytic terms, she was not dis-identifying from her mother, sister, and grandmother but rather negotiating which parts of them to integrate and which to refuse. Eventually, Sarika, too, had a chance to re-evaluate her identity in light of the changed circumstances. Indeed, disidentification helps an analysand when an internalized parental figure is a source of distress. However, it may mean foreclosing the parents’ positive aspects.

Therapy “uses the patient’s own conceptual framework to give new meaning to old situations […] freeing his [the patient’s] relationships from the rut in which they have gotten bogged down.” (Kakar, 1982, 277)

Kakar calls this the art of reinterpretation, one which, at St. Mary’s, the trainee must do to herself to begin with. Eventually, the affective practice that comes with training transforms her significant others. The doings of everyday life shape climates as much as climates shape and reproduce practices.

Eventually, Natasha created selfhood and collaborated to repair the fractured self of her mother after years of gendered moral injury. This is an ethic of self-making that is deeply embedded in kinship ties, yet oriented toward transforming the felt experience of those ties. She drew from psychological knowledge—attachment, trauma, emotional patterning—not to create distance from family, but to sustain proximity with greater clarity and intention. Her work was not about individuation but about transforming inherited pain into a livable ethical position. Here, the self was the medium through which changing emotional climate unfolded. With Sarika losing her fear of violence, Natasha’s fear of feeling unwanted by her mother was also addressed. Counseling training has the potential to constitute therapeutic capacity from the psychic afterlives of gender violence.

Counseling equipped trainees with language, concepts, and tools to revisit their familial experiences. Maternal figures in these narratives are not simply oppressive or nurturing but persistent carriers of affective intensities that shape psychic and moral development, not least of the children. The therapeutic encounter, in this case, becomes a means of reckoning with the intergenerational nature of gendered emotional climates rather than escaping them. In the Indian context, the maternal relationship is central to the formation of the self and often marked by both intense identification and profound ambivalence (Kakar, 2012). The classroom became a space to inhabit the ambivalence of this kinship.

## Discussion

The findings of this article argue against the assumption that counseling simply turns “Eastern” subjects into individualistic ones. This assumption is a dominant frame of reference accepted uncritically in counseling training syllabi (e.g., Baumeister & Bushman, 2017). Recent work has argued that psychotherapy cannot be reduced to a Eurocentric technology of the isolated self (Roy, 2025a) but can instead become a privileged site for rethinking the relation between person and society (Martin, 2025b). Natasha’s self-work does not detach her from kinship. It makes her more reflexively embedded in an emotional climate of mothering. Counseling, in this case, intensifies relational awareness rather than dissolving it into individualism.

This argument has direct clinical implications. It discourages treating maternal distress and filial suffering as discrete intrapsychic problems. Where a mother presents with “tension,” depression, volatility, or diffuse psychosomatic distress, or a daughter presents with anxiety, guilt, attachment insecurity, or overdeveloped responsibility, a clinician may do better to attend to their gendered emotional climate than to treat the distress as a discrete intrapsychic problem. In settings such as mine, trainees did not become therapeutic by separating from family life. This resonates with work on the “family assemblage,” which shows that mental health and recovery cannot be understood as purely personal processes, since they are constituted through the material, affective, and relational dynamics of family life (Price-Robertson et al., 2017).

This study also contains policy implications. In India, state responses to daughter devaluation have largely been organized through the idiom of demographic correction and development. Sex selection and the communication of fetal sex are legally prohibited, but the law has not been enough to address the perpetuation of son preference in spectacular and ordinary forms. The *Beti Bachao Beti Padhao* (BBBP, ‘Save Daughter Educate Daughter’) scheme has framed the problem in terms of declining child sex ratio, the prevention of gender-biased sex-selective elimination, and the survival, protection, and education of the girl child (Government of India, 1994; Ministry of Women & Child Development, 2019). Even where such interventions show demographic promise, as in evaluations of BBBP in Haryana, their governing grammar remains one of ratios, incentives, compliance, and behavioral change (Gupta et al., 2018). What such development-based interventions rarely address is the longer psychic and relational aftermath of gendered injury in mothers and daughters, both bound in a cycle of fear and loss. My findings, therefore, suggest that gender-justice interventions that stop at preventing the disappearance of girls risk neglecting the mental health afterlives of son preference, feticide, affinal violence, and maternal grief; incorporating family-sensitive, community-based mental health support into existing gender-policy frameworks could help address them.

## Conclusion

I have argued that gendered emotional climate names the mechanism through which structural gender violence is absorbed into domestic life and lived as the ordinary texture of kinship. The concept helps explain how suffering and healing move between generations through the enduring affective conditions of growing up: grief, subordination, the unequal distribution of care, and the spectral presence of fetuses not carried to term. Sex-selective abortion, like infant death, is not merely a biological or social event but a discursive and affective node through which power, care, and subjectivity are negotiated (Pinto, 2008). Natasha’s story suggests how counseling training in urban India can create conditions under which trainees reinterpret maternal suffering as the psychic afterlife of gendered violence rather than as individual maternal failure and can begin to alter the affective terms of kinship from within. In her case, training functioned as a form of relational repair, opening onto a more habitable gendered emotional climate.

Counseling equipped trainees with the language, concepts, and tools to revisit their experiences of kinship. Maternal figures in these narratives are not simply oppressive or nurturing but persistent embodiments of emotional climates that shape psychic and moral development, not least of the children. The therapeutic encounter, in this case, becomes a means of reckoning with the intergenerational nature of gendered harm.

## Data Availability

No datasets were generated or analyzed during the current study.

## Footnotes

1 I use the former name of the city ‘Bangalore’ rather than the current ‘Bengaluru’ introduced by the state of Karnataka in 2014 to reflect how my interlocutor’s referred to the city.

2 ‘Natasha’ is a pseudonym as are all other names of interlocutors as well as the name of the university ‘St Mary’s.’

3 The congregation that runs St Mary's belongs to a Christian minority in the predominantly Hindu state of Karnataka. St Mary’s is protected by the National Commission for Minority Educational Institutions (NCMEI) Act, 2004 and Article 30(1) of the Constitution of India. That gives them the legal right to admit a reasonable proportion of students from their own community while remaining secular public institutions. Thus, St Mary’s practices affirmative action for Christian students. Their postgraduate counseling curriculum is secular and clinical rather than catechetical, and the cohort includes trainees from Hindu, Christian, and other backgrounds. The self-work practices described here derive from humanistic and psychodynamic counseling traditions rather than Christian spiritual direction.

4 There is semantic overlap between counseling and therapy in India. Hence, I use the two terms interchangeably in this paper.

5 The two hours after recess every Thursday in this semester were reserved for the Professional Development (PD) course. The program coordinator granted me access to one of the four PD “batches” (groups). She led the batch I was part of. The objective of the course was to help trainees cultivate self-awareness and reflection—skills crucial to a career in counselling. Every week, students read out the reflective essays they had written about the themes assigned. The first theme was branching points, or moments that mark a critical juncture in a person’s development. The second theme was family.

6 Baniya refers to a merchant and trader caste concentrated in Haryana, Rajasthan, Gujarat, and other regions in northern and western India.

7 The term ‘core memory’ is drawn from the Pixar movie Inside Out, a cult favorite among the trainees. It is a metaphor derived from magnetic-core memory, a type of random-access memory (RAM); it signifes a formative memory that shapes someone’s personality.

8 Female feticide is common in Haryana, the Indian state with the lowest sex ratio, even though the Indian Penal Code prohibits it.

9 While ‘empathic’ is common in counseling pedagogy, Bella said ‘empathetic’ to refer to empathy as a general virtue.

10 See Karve (1953) for a detailed picture of North Indian kinship.
